# Microorganisms from Antarctica: A Review of Their Potential in the Bioremediation of Hydrocarbon-Contaminated Soils

**DOI:** 10.3390/microorganisms14050948

**Published:** 2026-04-22

**Authors:** Jaime Naranjo-Moran, María F. Ratti, Marcos Vera-Morales

**Affiliations:** 1 Grupo de Investigación en Aplicaciones Biotecnológicas, GIAB, Universidad Politécnica Salesiana, UPS, Carrera de Biotecnología, Campus María Auxiliadora, Kilómetro 19.5 Vía a La Costa, Guayaquil 090901, Ecuador; 2 Centro de Investigaciones Biotecnológicas del Ecuador, CIBE, Escuela Superior Politécnica del Litoral, ESPOL, Campus Gustavo Galindo Km. 30.5 vía Perimetral, Guayaquil 090112, Ecuador; mratti@espol.edu.ec

**Keywords:** bioremediation, cold-active enzymes, psychrophiles, metagenomics, bioavailability, Antarctic Treaty

## Abstract

Antarctica’s extreme cryospheric conditions impose severe thermodynamic constraints on the natural attenuation of hydrocarbon pollutants. Despite the Antarctic Treaty System’s protections, the footprint of human logistics has left persistent reservoirs of petroleum hydrocarbons that threaten endemic biodiversity. This review critically synthesizes the state-of-the-art in Antarctic bioremediation, moving beyond traditional culture-dependent studies to integrate recent multi-omics breakthroughs (2020–2025). We analyze the molecular mechanisms limiting bioavailability in frozen soils and highlight the adaptive strategies of psychrophilic consortia, including the modification of membrane fluidity and the expression of cold-active enzymes (e.g., RHDs, AlkB). Notably, we discuss emerging findings on novel long-chain alkane degradation genes (*almA*, *ladA*) identified in 2025, which challenge previous assumptions about recalcitrance. Furthermore, the review evaluates the engineering bottlenecks of in situ versus ex situ strategies, emphasizing the synergistic potential of bacterial–fungal co-cultures and the ecological necessity of “climate-smart” remediation to mitigate methane emissions from thawing permafrost. By bridging the gap between fundamental microbial genetics and applied field engineering, we propose a roadmap for the next generation of biotechnological solutions in the warming polar environment.

## 1. Introduction

Antarctica, the most isolated and southernmost continent on Earth, is characterized by perennial freezing temperatures and extensive ice cover that challenge the limits of biological existence [1]. Despite international conservation efforts governed by the Antarctic Treaty System [2], the region’s pristine status is increasingly threatened by anthropogenic stressors [3,4]. Over the last decade, the exponential growth in scientific logistics, research stations, and tourism has intensified the risk of environmental contamination [5]. Among the various pollutants, petroleum-derived hydrocarbons remain the most predominant and persistent threats to Antarctic soils [6,7]. The scale of this issue is significant; historical estimates suggest that between 1 and 10 million m^3^ of soil in Antarctica are contaminated with petroleum products [8]. These spills, often resulting from fuel storage and transportation, are particularly severe in coastal ice-free areas where biodiversity is concentrated [9]. In these extreme environments, low temperatures drastically increase hydrocarbon viscosity and reduce volatilization, leading to long-term persistence in the subsurface, which directly affects the native flora and macrofauna [10,11].

While physical and chemical remediation is often logistically nonviable or ecologically disruptive, bioremediation harnesses the metabolic versatility of microorganisms to mineralize these pollutants [12,13]. Indigenous psychrophilic and psychrotolerant microorganisms—including bacteria like *Pseudomonas* and *Rhodococcus* and fungi such as *Penicillium*—have evolved sophisticated molecular adaptations to thrive in the extreme conditions of Antarctica [14]. These strategies involve the modification of membrane fluidity via unsaturated fatty acids [15], the synthesis of cryoprotectants like trehalose and polyamines [16], and the production of cold-active enzymes (e.g., monooxygenases, lipases, and laccases) capable of degrading aliphatic and aromatic compounds at sub-zero temperatures. Despite the great potential of these microorganisms, traditional perspectives on Antarctic bioremediation have often been limited to the description of cultivable diversity [17]. Recent advances in 2024 and 2025 have shifted the focus toward multi-omics approaches (metagenomics, metabolomics, and proteomics) to unravel the complex interactions within microbial consortia that drive degradation efficiency [18]. Current research highlights the synergistic potential of bacterial–fungal co-cultures and the critical role of biosurfactants in overcoming bioavailability bottlenecks in frozen soils [19].

Therefore, this review aims to provide an update of the status of hydrocarbon bioremediation in Antarctica. Unlike previous reviews, we integrate recent findings on molecular adaptation mechanisms, the emerging role of “uncultivable” microbial fractions revealed by metagenomics, and the latest biotechnological strategies to improve remediation rates in the polar cryosphere.

**Part I: Pollution context and challenges**

## 2. Antarctic Pollution

***Hydrocarbon contamination landscape in the Antarctic cryosphere***

While the Antarctic Treaty System designates the continent as a natural reserve devoted to peace and science [2], the footprint of human activity has become indelible in specific locations. The contamination landscape is primarily driven by the logistical reliance on fossil fuels for energy generation, heating, and transport operations, which are essential for survival in polar conditions. Unlike temperate regions, the Antarctic environment presents a unique “chemical paradox”: while photooxidation can degrade surface spills during the austral summer, the extreme cold and ice cover “freeze” contaminants in the subsurface, preserving their toxicity for decades [20].

### 2.1. Sources and Distribution

Hydrocarbon pollution is not uniformly distributed but is patchily concentrated around the ~76 active research stations and high-traffic coastal areas [21]. The primary pollutants are Special Antarctic Blend (SAB) diesel and marine gas oil, which are engineered to remain liquid at low temperatures but possess high mobility in porous soils. Recent assessments in 2024 indicate that even in “pristine” areas, atmospheric deposition and long-range transport contribute to the background contamination, complicating the definition of clean control sites [20]. Moreover, the increasing frequency of tourist expeditions adds a new layer of risk regarding accidental marine spills and the translocation of hydrocarbons on microplastic surfaces [7].

### 2.2. Chemical Recalcitrance and Bioavailability

The fundamental challenge in Antarctic bioremediation is not merely the presence of hydrocarbons but their bioavailability at low temperatures. The high viscosity of petroleum products reduces the mass transfer rates of substrates into microbial cells [6]. For example, n-Alkanes (C9–C15), the main components of diesel, are generally susceptible to microbial attack. However, as the fuel “ages” in the soil, the lighter, more volatile fractions evaporate, leaving behind heavier, hydrophobic chains that bind tightly to soil organic matter, becoming inaccessible to microbial enzymes. Research from 2023 demonstrates that this “aged” diesel becomes significantly more toxic to endemic soil nematodes compared to fresh spills, likely due to the persistence of recalcitrant polar metabolites resulting from partial photo-oxidation [22]. Aromatic complexity: polycyclic aromatic hydrocarbons (PAHs) such as naphthalene, phenanthrene, and their alkylated derivatives represent the most toxic fraction. Recent studies on the Fildes Peninsula have highlighted that these compounds are not only persistent but are also undergoing trophic transfer, accumulating in the local food web from soil invertebrates to penguins [23,24]. This implies that remediation strategies must address not just the parent hydrocarbons but also the complex mixture of weathered byproducts that traditional TPH (total petroleum hydrocarbon) analyses often overlook.

## 3. Hydrocarbons

***Chemical Profile and Biological Fate of Antarctic Hydrocarbons***

The pollutants found in Antarctic soils are not crude oils but refined middle-distillate fuels, primarily classified into paraffins (linear and branched alkanes), naphthenes (cycloalkanes), and aromatics [25]. The predominant fuel used in the region, SAB, is characterized by a high proportion of n-alkanes (C9–C15) and a reduced content of heavier waxes to prevent freezing. However, in the event of spillage, this low viscosity facilitates rapid vertical migration through the porous active layer toward permafrost or phreatic zones, significantly impacting subsurface bacterial community structures [26].

### 3.1. The Aromatic Challenge

While aliphatic fractions constitute the bulk of the contaminant mass, the environmental risk is disproportionately driven by the aromatic fraction. This group includes monocyclic compounds (BTEX: benzene, toluene, ethylbenzene, and xylene) and PAHs such as naphthalene and methylnaphthalenes [6]. The presence of these compounds is critical because, unlike aliphatics, many PAHs—specifically styrene and naphthalene—are classified by the IARC as Group 2B carcinogens, underscoring a severe ecotoxicological concern even at low concentrations [27]. Furthermore, recent studies confirm that while bacteria may rapidly deplete linear alkanes, the remaining aromatic residues can inhibit microbial growth and reduce community diversity to a greater extent than aliphatic contamination alone [28].

### 3.2. Microbial Selection and Metabolic Potential

The Antarctic environment imposes the persistence of aromatic compounds by restraining their bioavailability. The interaction between soil microbiota and contaminants is bidirectional. On the one hand, hydrocarbons exert a strong selective pressure, enriching the soil with specific degraders while suppressing sensitive taxa. On the other hand, the metabolic activity of these enriched communities actively modifies the contaminants through mineralization or transformation into less toxic intermediate compounds, thereby altering the chemical profile of the soil.

- **Recalcitrance:** Aromatic rings provide thermodynamic stability that resists microbial attack, requiring specialized enzymatic machinery (e.g., ring-hydroxylating dioxygenases) that is metabolically expensive to produce in nutrient-scarce, sub-zero conditions.
- **Consortia Efficiency:** Consequently, single-strain degradation is rarely sufficient. Current research emphasizes the necessity of microbial consortia—associations of bacteria and fungi—to achieve broad-spectrum remediation. Under controlled conditions, optimized consortia have demonstrated the capacity to mineralize up to 98% of total petroleum hydrocarbons (TPH), although field rates are often lower due to abiotic limitations [19,29].

**Part II: Microbial mechanisms (bacteria, fungi, consortia)**

## 4. Bacterial Strategies for Hydrocarbon Degradation in Antarctic Soils

Effective mitigation of hydrocarbon contamination in the cryosphere demands tailored bioremediation strategies anchored in a deep understanding of microbial physiology. Bacteria in Antarctic soils are not merely resistant; they are metabolically adapted psychrophiles and psychrotolerants capable of driving biogeochemical cycles under extreme stress [15]. Antarctic soils harboring hydrocarbon contamination select for specialized heterotrophic populations capable of utilizing these substrates as their primary carbon and energy source [12]. However, the presence of these bacteria does not guarantee remediation; their degradation efficiency is governed by a complex interplay of abiotic stressors (e.g., salinity, freeze–thaw cycles) and biotic mechanisms (e.g., biofilm formation, biosurfactant production). Table 1 summarizes recent findings on key bacterial isolates and the specific molecular or physiological mechanisms that facilitate hydrocarbon biotransformation in these environments.

### 4.1. Bacterial Diversity and Metabolic Versatility

Antarctic bacteria associated with hydrocarbon contaminants exhibit metabolic versatility, capable of degrading aliphatic fractions [31], aromatic hydrocarbons [32], and recalcitrant polycyclic compounds like phenanthrene [29]. The dominant genera in these soils—*Pseudomonas*, *Rhodococcus*, and *Sphingomonas*—have evolved distinct survival strategies. *Rhodococcus* species are particularly effective due to their cell surface hydrophobicity and biosurfactant production, which allow them to adhere directly to hydrocarbon droplets and degrade long-chain alkanes (n-dodecane and crude oil) at low temperatures [30]. Meanwhile, *Sphingomonas* strains demonstrate exceptional resilience to environmental stressors, tolerating high UV radiation levels and repeated freeze–thaw cycles while metabolizing complex organics [32]. Other psychrotolerant genera, including *Arthrobacter* and *Janthinobacterium*, have recently been identified as key players in diesel degradation, utilizing the fuel as a sole carbon source via synergistic interactions in microbial consortia [14,31].

### 4.2. Cold-Adapted Enzymatic Pathways

**Figure 1:**
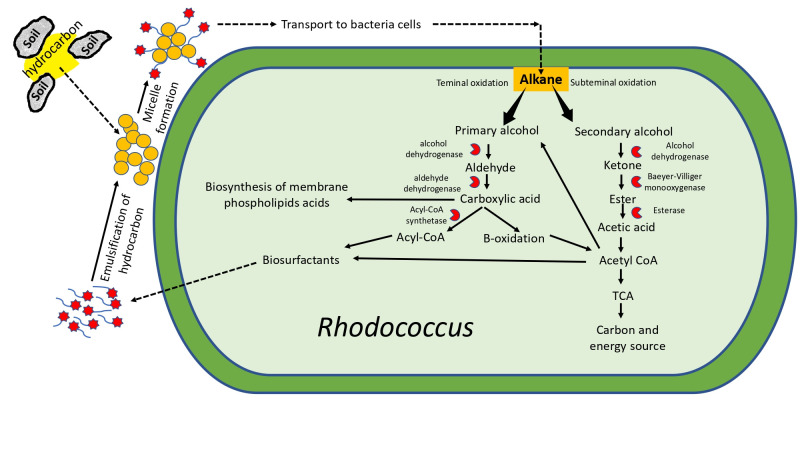
Degradation of alkane aliphatic hydrocarbons by the psychrophilic bacterium *Rhodococcus*.

The degradation of aliphatic alkanes in Antarctic bacteria is driven by specialized enzyme systems that overcome the thermodynamic barriers of sub-zero environments. The primary mechanism involves cold-active monooxygenases (e.g., AlkB and cytochrome P450), which initiate oxidation by introducing oxygen into the chemically inert alkane chain [33]. Unlike their mesophilic counterparts, these Antarctic enzymes have structural adaptations—such as increased flexibility around the active site—that maintain catalytic efficiency at low kinetic energy states [34]. The pathway typically proceeds through terminal or subterminal oxidation: the monooxygenase, coupled with electron carriers (rubredoxin/rubredoxin reductase), converts the alkane into a primary or secondary alcohol. This intermediate is subsequently oxidized by alcohol and aldehyde dehydrogenases into fatty acids, which enter the beta-oxidation cycle to generate Acetyl-CoA for the TCA cycle [35]. Figure 1 illustrates this pathway, highlighting the crucial role of membrane fluidity modifications that facilitate the transport of hydrophobic substrates to membrane-bound enzymes like AlkB.

### 4.3. Catabolism of Recalcitrant Aromatic Hydrocarbons

PAHs represent a significant challenge in Antarctic soils due to the thermodynamic stability of their fused benzene rings and the absence of terminal methyl groups, which limits the attack sites for microbial enzymes [10]. While bioremediation data in cryospheric environments predominantly focuses on aerobic pathways, the metabolic versatility of psychrophiles is broader than previously understood.

#### 4.3.1. Aerobic Ring-Cleavage Pathways

**Figure 2:**
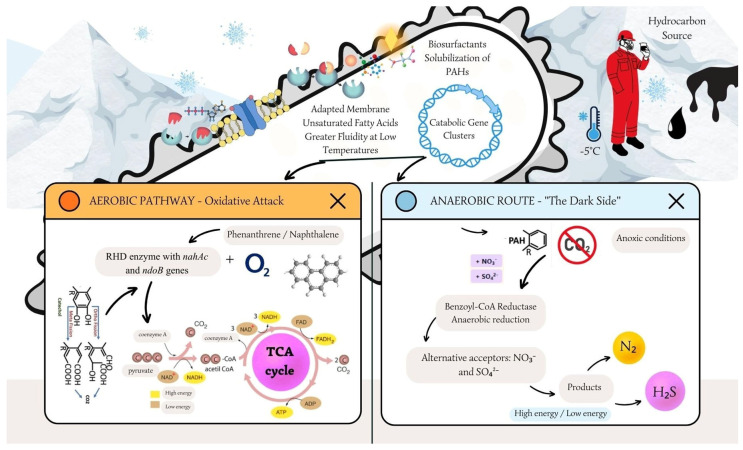
Schematic representation of aerobic and anaerobic hydrocarbon degradation pathways in an Antarctic psychrophilic bacterium.

Under aerobic conditions, the degradation of PAHs is initiated by ring-hydroxylating dioxygenases (RHDs). These multicomponent enzyme systems catalyze the incorporation of molecular oxygen into the aromatic nucleus to form *cis*-dihydrodiols [36]. Unlike mesophilic enzymes, Antarctic variants of RHDs (encoded by genes such as *nahAc* and *ndoB*) exhibit lower activation energies, allowing catalysis to proceed at near-zero temperatures [32]. Subsequent dehydrogenation yields dihydroxylated intermediates (e.g., catechol or protocatechuate), which serve as central metabolic hubs. From this junction, ring cleavage occurs via either ortho-cleavage (yielding succinate/acetyl-CoA) or meta-cleavage (yielding pyruvate/acetaldehyde), ultimately leading to mineralization (CO_2_ and H_2_O). While the dioxygenase pathway is dominant in proteobacteria like *Pseudomonas*, other key Antarctic genera have alternative routes. For instance, *Mycobacterium* species produce a cytochrome P450 monooxygenase system to oxidize PAHs into trans-dihydrodiols, a pathway analogous to eukaryotic metabolism [37]. Additionally, specific psychrotolerant strains of *Colwellia* and *Roseovarius* have been found to degrade cyclic alkanes (e.g., methylcyclohexane) via a complementary lactone-formation pathway, demonstrating a high degree of metabolic specialization in niche Antarctic environments [38]. Figure 2 illustrates these divergent pathways, highlighting the specific gene clusters identified in recent Antarctic metagenomes.

#### 4.3.2. The Anaerobic Frontier

A critical yet understudied aspect of Antarctic bioremediation is anaerobic degradation. Subsurface soils and permafrost layers are often anoxic. Although research is limited, recent evidence identifies specific genera—such as *Geobacter* and *Thiobacillus*—capable of coupling hydrocarbon oxidation to nitrate or sulfate reduction at temperatures as low as −5 °C [39]. Unlocking these anaerobic mechanisms is crucial for in situ remediation of deep, frozen soil horizons where oxygen diffusion is physically restricted.

Under aerobic conditions (left), RHDs encoded by genes such as *nahAc* initiate the attack using molecular oxygen, channeling intermediates via catechol into the TCA cycle. Under anaerobic conditions (right), alternative electron acceptors (NO_3_^−^, SO_4_^2−^) drive the oxidation via benzoyl-CoA reductases. The diagram emphasizes the role of membrane modifications and biosurfactant-mediated uptake in overcoming mass transfer limitations at low temperatures.

### 4.4. Overcoming Bioavailability Barriers: Biosurfactants and Biofilms

In the Antarctic subsurface, hydrocarbon biodegradation rate is reduced by their low diffusion into aqueous phases. To overcome this, psychrotolerant bacteria rely on two primary physical strategies:

- **Biosurfactant Production:** Bacteria secrete amphipathic molecules—classified into glycolipids (rhamnolipids, sophorolipids), lipopeptides, and phospholipids—that reduce surface tension at the oil-water interface [40]. Recent studies in 2024 highlight that Antarctic strains of *Pseudomonas* and *Janthinobacterium* can achieve emulsification indices (E_24_) exceeding 60% even at 4 °C, creating “pseudosolubilized” micelles that facilitate active uptake via membrane channels [14]. Unlike synthetic surfactants, these biomolecules remain stable and active despite extreme salinity and temperature fluctuations [41].
- **Biofilm Formation:** Rather than interacting with solubilized fractions, some genera like *Rhodococcus* exploit their hydrophobic cell surfaces (rich in mycolic acids) to adhere directly to hydrocarbon droplets. This attachment is often regulated by quorum sensing signaling molecules, which trigger the formation of intricate biofilms. These biofilms protect the community from osmotic stress and freeze–thaw cycles while creating a catalytic microenvironment where exoenzymes are concentrated against the pollutant surface [30].

### 4.5. Key Genera and Ecological Niches

While metagenomic surveys reveal a vast uncultured diversity, three genera consistently emerge as the “keystone engineers” of Antarctic bioremediation due to their distinct ecological roles:

- ***Rhodococcus*** (The Generalist): Renowned for its metabolic robustness, *Rhodococcus* utilizes a vast array of aliphatic and aromatic substrates. Its distinct advantage lies in its cell envelope hydrophobicity and the ability to produce trehalose lipids, which function as both cryoprotectants and biosurfactants [31].
- ***Pseudomonas*** (The Genetic Reservoir): Ubiquitous in contaminated polar soils, this genus is characterized by high genomic plasticity. It frequently harbors catabolic plasmids (e.g., TOL, NAH) encoding degradative pathways for complex aromatics, coupled with strong biofilm-forming capabilities driven by cold-adapted exopolysaccharides [14].
- ***Sphingomonas*** (The Aromatic Specialist): This genus is critical for the degradation of recalcitrant PAHs. Recent genomic analyses (2023) have identified specific gene clusters in Antarctic *Sphingomonas* that allow for the cleavage of fused ring systems (like carbazole) under high UV radiation and oxidative stress conditions typical of the Antarctic surface [32].

Other emerging players such as *Mycobacterium*, *Gordonia*, and *Aeromicrobium* are increasingly recognized for their role in alkane degradation, though their ecological distribution appears more dependent on specific soil physicochemical properties such as pH and nitrogen availability [26].

### 4.6. Future Perspectives: From Strains to Synthetic Consortia

The era of isolating single strains for bioremediation is evolving into an era of microbial community engineering. Future research must shift focus from individual metabolic characterization to understanding the synergistic interactions within natural and synthetic consortia. The most effective degradation of complex hydrocarbon mixtures—particularly those containing recalcitrant PAHs—occurs when fungal enzymes initiate oxidative attack, creating intermediates for bacterial mineralization [19]. A critical bottleneck remains the “unculturable” majority. The application of metagenomics and metatranscriptomics is crucial to identify novel cold-active enzymes and regulatory networks that do not function in standard laboratory cultures [18]. Furthermore, advancing synthetic biology tools to engineer psychrophilic consortia with enhanced biosurfactant production or optimized metabolic cross-feeding could achieve Antarctic sustainable and highly efficient in situ bioremediation.

## 5. Fungal Remediation in the Cryosphere

While bacteria are often the first responders to aliphatic spills, fungi play an indispensable role in the degradation of high-molecular-weight and recalcitrant aromatic compounds. In the Antarctic cryosphere, fungi possess a distinct ecological advantage over bacteria: their hyphal extension allows them to penetrate frozen, low-permeability soil matrices, effectively translocating water and nutrients to the contamination front [42]. Furthermore, their adaptation to high UV radiation and desiccation involves the production of melanin and cryoprotective sugars (trehalose), mechanisms that incidentally enhance their resilience to chemical toxicity [43].

### 5.1. Enzymatic Mechanisms: Beyond Intracellular

Unlike bacteria, which often require substrate internalization, Antarctic filamentous fungi secrete powerful extracellular ligninolytic enzymes—such as laccases, manganese peroxidases (MnP), and lignin peroxidases (LiP). These non-specific oxidative enzymes generate free radicals that can attack the stable ring structures of PAHs (e.g., anthracene, pyrene) and complex aliphatic chains via co-metabolism [44]. Recent studies in 2023 have isolated strains of *Penicillium* from Antarctic soils able to degrade complex hydrocarbons, not as a sole carbon source, but through co-metabolic oxidation driven by high-redox-potential enzymes [45]. Additionally, the intracellular cytochrome P450 monooxygenase system in fungi serves as a primary detoxification route, converting PAHs into trans-dihydrodiols (in contrast to bacterial *cis*-dihydrodiols), which are less toxic and more water-soluble [46] (Table 2).

### 5.2. The Role of Psychrotolerant Yeasts

Often overlooked, Antarctic yeasts are emerging as critical biotechnological tools. Genera such as *Candida*, *Rhodotorula*, and *Pichia* are not only capable of metabolizing alkanes but are also prolific producers of extracellular biosurfactants (e.g., mannosylerythritol lipids). These compounds significantly enhance the bioavailability of hydrophobic pollutants in the aqueous phase of melting ice, creating a synergistic effect when coupled with bacterial degradation [19,50].

### 5.3. Fungal Resilience and Enzymatic Promiscuity

While hydrocarbon contamination exerts a strong selective pressure that inhibits sensitive fungal taxa, it simultaneously favors the proliferation of resilient “weed” species capable of exploiting xenobiotics. In Antarctic soils, this is evident in the dominance of genera such as *Phialophora* and *Pseudogymnoascus* in heavily contaminated sites [49]. Unlike bacteria, whose monooxygenases are often highly substrate-specific, filamentous fungi deploy ligninolytic enzymes (laccases and peroxidases) that exhibit low substrate specificity. This “enzymatic promiscuity” allows them to oxidize complex aromatic rings (e.g., benzo[a]pyrene) via free radical mechanisms, bypassing the need for specific membrane transporters [43].

Recent investigations confirm that this capability is widespread among Antarctic isolates. For instance, strains of *Penicillium* have demonstrated the ability to degrade a broad range of aliphatic (n-octane to n-dodecane) and aromatic hydrocarbons (ethylbenzene, naphthalene). Similarly, the psychrotolerant fungus *Aspergillus glaucus* uses naphthalene and anthracene as sole carbon sources by producing intracellular catechol dioxygenases similar to bacterial pathways.

### 5.4. The Untapped Potential of Psychrophilic Yeasts

Beyond filamentous fungi, Antarctic yeasts represent a largely untapped resource for biotechnology. Their unicellular nature allows for rapid growth and high-surface-area contact with emulsified hydrocarbons.

- **Black Yeasts:** Species like *Exophiala macquariensis* possess melanized cell walls that provide exceptional protection against UV radiation and oxidative stress while degrading volatile aromatics like toluene.
- **Biosurfactant Producers:** Comprehensive screenings of Antarctic yeasts have identified genera such as *Candida*, *Metschnikowia*, and *Rhodotorula* as prolific degraders of n-hexadecane and phenol. Notably, *Pichia caribbica* has been identified for its high esterase and lipase activity, crucial for the breakdown of diesel fuel lipids.

These findings suggest that yeasts may play a pivotal role in the initial emulsification of pollutants, facilitating subsequent bacterial mineralization in mixed-consortia applications.

### 5.5. Fungal–Bacterial Consortia in Hydrocarbon Degradation

Synergistic interactions between fungi and bacteria represent a powerful biological tool for Antarctic bioremediation, harnessing fungal mycelial networks to enhance pollutant bioavailability and bacterial mobility within the soil. In addition, multiple biological domains exhibit complex relational dynamics and emergent properties. Within these mixed-species co-cultures, bacteria and fungi often develop synergistic metabolic networks, characterized by the synchronized secretion and complementary action of diverse extracellular enzymes [51]. Evidence suggests that while *Penicillium janthinellum* is incapable of independent PAH mineralization, its cocultivation with *Stenotrophomonas maltophilia* significantly accelerates the degradation of compounds such as chrysene and benz[a]anthracene [52]. The physical architecture of filamentous fungi acts as a conduit for the transport of hydrophobic pollutants, thereby increasing their bioavailability to indigenous microbes [53,54].

Success in mixed-strain remediation efforts comes from synergistic effects that exceed the capabilities of individual isolates. Previous research involving *Sarocladium* and *Cryptococcus* consortia highlights how simultaneous biosurfactant production and hydrocarbon breakdown are optimized through such partnerships. This efficiency is attributed to a division of metabolic labor, where the diverse components of crude oil are partitioned and processed by different members of the co-culture [55]. Moreover, microbial consortia facilitate the breakdown of PAH mixtures compared to single-strain applications. By immobilizing these populations on organic matrices like corn cobs, researchers have proven more stability in the field. This carrier-based approach acts as a physical shield against fluctuating conditions, ensuring that the consortium retains its functional capacity [56]. Biochar has also been used as a carrier to fix microorganisms and improve their activity and survival rate, increasing the removal effect by more than 10% higher than free microorganisms [57]. A mix of *Pseudomonas* sp. S4 and *Mortierella alpina* J7 was used with biochar to degrade PAHs (phenanthrene and pyrene), facilitating the transition of PAHs from an adsorbed state to a microbially accessible one. This carrier maximized the synergistic metabolic potential of the consortium [58]. Despite the limitations of low temperature and scarce previous experiences in situ, the integration of multi-strain consortia represents a robust strategy for hydrocarbon degradation in extreme polar environments.

**Part III: Engineering and field applications**

## 6. Engineering Remediation in the Cryosphere: Strategies and Limitations

Implementing bioremediation in Antarctica presents logistical challenges unparalleled in temperate regions. The choice between in situ and ex situ strategies is dictated not only by the extent of contamination but also by the preservation of the delicate permafrost thermal regime and the “active layer” depth—the soil horizon that thaws during austral summers [11]. In this context, Table 3 summarizes and compares the main in situ and ex situ bioremediation strategies applied in Antarctica, highlighting their operational advantages and limitations, as well as the results obtained from representative case studies.

### 6.1. In Situ Limitations: The Bioavailability Bottleneck

In situ approaches, such as bioventing and biostimulation, are generally preferred for minimizing physical ecosystem disturbance [6]. However, field trials at major research stations (e.g., Casey, Marambio) have demonstrated that efficacy is strictly limited by abiotic factors. Beyond the increase in hydrocarbon viscosity, low increases in soil temperature reduce oxygen solubility, while the underlying permafrost acts as an impermeable barrier, often causing waterlogging and anaerobic conditions in the active layer during thaw periods [61]. Consequently, natural attenuation alone is often insufficient for aged spills.

Biostimulation, the addition of rate-limiting nutrients (N and P), has proven effective, but stoichiometry is critical. Recent long-term studies indicate that while optimized C:N:P ratios can enhance alkane removal by up to 75% in 50 days, excessive fertilizer application can induce osmotic stress in oligotrophic Antarctic microbial communities, diminishing degradation rates. Furthermore, the use of slow-release fertilizers (e.g., oleophilic fertilizers) is recommended to prevent rapid nutrient leaching into pristine glacial meltwaters [60].

### 6.2. Ex Situ Efficiency: Biopiles and Bioaugmentation

**Figure 3:**
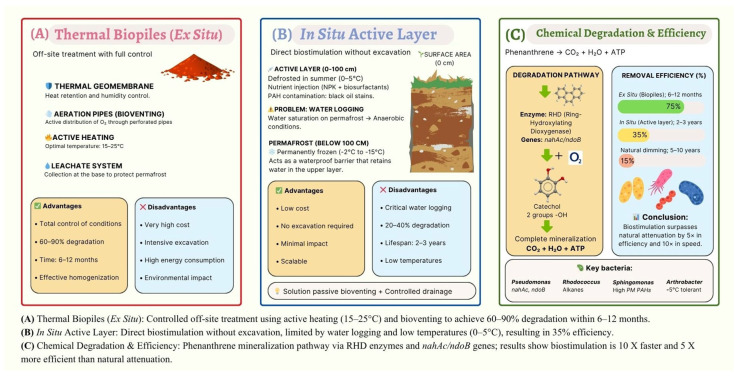
Bioremediation strategies for PAH-contaminated Antarctic soils: ex situ vs. in situ approaches.

For high-concentration hotspots, ex situ treatments like biopiles offer greater control over environmental parameters. The construction of biopiles with high-density polyethylene geomembranes and active aeration systems allows for the manipulation of temperature and moisture, extending the window of microbial activity beyond the short austral summer [60]. Furthermore, the incorporation of fungal–bacterial consortia into these systems enhances soil porosity, and the hyphal networks serve as scaffolds for the distribution of extracellular enzymes, making the degradation possible in poorly oxygenated zones of the soil matrix. For example, the aggregate hierarchy hypothesis with life cycle models highlights the dual role of arbuscular mycorrhizal fungi. Hyphae first mechanically enmesh macro-aggregates (250–2500 μm) before producing glomalin-related proteins that bind micro-aggregates (<250 μm) internally. This sequential process ensures long-term soil stability and creates a structured environment for microbial activity [62]. Another factor to consider is bacterial chemotaxis as a critical driver for naphthalene and PAH degradation. The efficacy of this activity can be hindered by the lack of liquid films. But filamentous fungi overcome this by developing mycelial networks that breach air-water interfaces and bridge saturated and unsaturated soil pores. Thus, fungi facilitate the dispersal of bacteria and the translocation of nutrients across heterogeneous systems, connecting degrading organisms with isolated pollutant patches that would otherwise remain unavailable [63]. Notably, recent research has re-evaluated bioaugmentation. While the Protocol on Environmental Protection to the Antarctic Treaty strictly regulates the introduction of non-native taxa, the enrichment and re-introduction of indigenous consortia (e.g., pre-adapted *Pseudomonas* sp.) has achieved removal efficiencies of 81–100% for diesel range organics in controlled field plots [64,65]. This “autochthonous bioaugmentation” represents a legally viable strategy to accelerate the lag phase of degradation (Figure 3, Table 4).

## 7. Biotechnological Frontiers: Cold-Active Enzymes and Biosurfactants

The Antarctic cryosphere is not merely a sink for contaminants but a reservoir of unique genetic resources. Beyond biomass, the focus of current research has shifted to extracellular products—specifically cold-active enzymes (CAZymes) and biosurfactants—that drive remediation at thermodynamic extremes.

### 7.1. Cold-Active Enzymes: Overcoming the Kinetic Barrier

Standard enzymes lose significant catalytic efficiency as temperatures approach 0 °C due to reduced molecular kinetic energy. However, Antarctic microorganisms produce psychrophilic enzymes (e.g., lipases, proteases, and alkane hydroxylases) characterized by high structural flexibility around the active site and reduced activation energy [33]. This allows them to maintain turnover rates (k cat) at 4 °C comparable to mesophilic enzymes at 25 °C. For instance, recent screenings of *Arthrobacter* and *Rhodococcus strains* have involved isolating oxidases capable of initiating hydrocarbon attack at temperatures as low as −5 °C, a critical trait for in situ bioremediation during the Antarctic shoulder seasons [31,34].

### 7.2. Biosurfactants: Thermodynamics at Freezing Points

Antarctic bacteria overcome the low viscosity of oils by secreting cold-active biosurfactants (CABSs). Unlike synthetic surfactants (e.g., SDS), which may precipitate or lose activity in cold/saline water, CABSs, like rhamnolipids and sophorolipids, maintain their critical micelle concentration stability under freeze–thaw conditions [41]. Mechanistically, these amphipathic molecules accumulate at the oil–water interface, reducing surface tension from ~72 mN/m to <30 mN/m. This induces the pseudosolubilization of hydrocarbons into stable micelles, drastically increasing the contact surface area for microbial uptake [40]. Recent studies (2024) highlight the potential of genera such as *Janthinobacterium* and *Pseudomonas fildesensis*, which produce glycolipid biosurfactants at 4 °C with emulsification indices (E_24_) ranging from 36.4% to 66.7%. Crucially, these values are comparable to or exceed those of commercial chemical surfactants, validating their potential for developing “green” dispersants for spill response in polar waters [14].

**Part IV: Future directions (omics, climate adaptation)**

## 8. Limitations and Outstanding Challenges

### 8.1. From Lab to Field: The Scaling Gap

Soil characteristics, such as mineral composition and nutrient profiles, are drivers of microbial activity. By influencing metabolic rates, these factors determine which taxa successfully colonize soils, defining the resulting community assembly [66]. In addition to those factors, changes in humidity, pH, and thermal gradients that can shift abruptly force the survival mechanisms of indigenous microflora to evolve across these ranges. Because these habitat conditions are heterogeneous even at the micrometer level, they result in shifts in the fluctuation of microbial population structures [67]. In contrast to controlled conditions, where stable parameters govern the environment, in the field, the same microorganisms respond differently to hydrocarbon pollutants. Their performance in natural settings remains constrained by ecological stressors and site-specific dynamics. To address these variations, there are several alternatives:

Martinez et al. (2020) assayed different factors hindering bioremediation in the field, determining that the C:N:P ratio needed pre-optimization to be successful in both scenarios [68]. Additionally, simulation experiments mimic the climatic conditions of Antarctica and other environments with shifting parameters using equipment like simulation chambers (currently adapted to studies in bacteria). Another approach is to design a combination of laboratory and in situ experiments whose results are more realistic in response to the harsh conditions on the White Continent [69]. The robustness of any bioremediation approach must be validated by identifying the factors that impact reproducibility during field trials. Because these processes are biological, their success is modulated by site-specific physicochemical variables [68]. Thus, field trials are still limited to the validation of models for in situ bioremediation; therefore, all efforts should be made with a prior site-specific study and meticulous posterior monitoring [11].

### 8.2. The “Unculturable Majority” and Molecular Access

Even in the era of omics studies, soil polar compartments are difficult to understand, and the results are mostly not comparable among sites, so the distribution of Antarctic microorganisms is still far from being resolved [70]. There are extensive studies assessing the potential of microbial strains to bioremediate cold/polar environments; however, these processes rely not only on the individual capacity of the microorganisms involved but also on the interactions among their communities. In addition, most members of the environmental microbiomes are not culturable, and their functions remain obscure [17]. Despite the advent of new technologies to culture strains, the complex characteristics of natural environments, with the additional limitations of unique and low-nutrient conditions of polar soils [71]. Molecular approaches have been developed to analyze both the taxa and the functionality of these microorganisms. Mining metagenomic data has proven to be an important tool for the annotation of aromatic-ring hydroxylating oxygenase genes, which may be involved in PAH degradation by unculturable microbes [17].

Several genes are involved in PAH degradation or transformation and seem to be upregulated in controlled experiments at medium to low temperatures. Previous studies show that dioxygenase, naphthale 1,2-dioxygenases can catalyze PAHs by producing hihydroxyl structures. Other genes related to PAH transformation are aldehyde dehydrogenase (*nidD*), 4-(2-carboxyphenyl)-2-oxobut-3-enoate aldolase (*phdJ*), and hydratase-aldolase (*phdG*) [72]. Furthermore, genomic investigations into psychrotolerant *Pseudomonas fluorescens* have identified specialized genes responsible for channeling PAH terminal metabolites and heterocyclic derivatives into central metabolic pathways. This strain also exhibits a high abundance of detoxification-related genes, including glutathione S-transferase and various quinone oxidoreductases. The survival of these psychrotolerant microbes is supported by a genetic repertoire: while one set governs hydrocarbon catabolism, a secondary suite of genes (encoding fatty acid desaturases, cryoprotectants, and pigments) ensures physiological resilience under sub-zero conditions [73]. Nevertheless, we need to consider that different species respond uniquely to diverse environmental parameters, and the family of genes involved also changes accordingly. To better understand the microbial metabolism of PAHs, researchers have leveraged transcriptomics and metabolomics tools to map specific degradation pathways. These high-throughput studies identify the essential catabolic genes and enzymes responsible for pollutant breakdown, serving as a foundational resource for evaluating the efficacy of microbial communities as bioremediators. Genetic studies on the bioremediation of PAHs are limited to low-molecular-weight PAHs (two to three rings), and investigations on high-molecular-weight PAHs (four or more rings) are still limited. Taking into consideration that PAHs are found as a mixture in the environment, genetic analyses seem to be required for high-molecular-weight PAH degradation [74].

### 8.3. Bioaugmentation vs. Biostimulation: The Efficiency Paradox

A debate persists in Antarctic literature regarding the most effective strategy for accelerated bioremediation: biostimulation or bioaugmentation. While biostimulation is often considered safe and feasible, it is limited to nutrient availability. In contrast, bioaugmentation introduces highly fitted degraders to act on persistent pollutants. However, its implementation in Antarctica faces an efficiency paradox: introduced strains often suffer from low fitness when faced with the extreme fluctuating temperatures and competition with established local biofilms [75]. Furthermore, the Antarctic Treaty System imposes strict legal barriers on the introduction of non-native species, making bioaugmentation a regulatory challenge. Future research must bridge this gap by exploring autochthonous bioaugmentation (if possible) to bypass both biological rejection and legal constraints, determining whether the added cost and complexity of inoculation truly outweigh the simplicity of nutrient amendments [76].

Sustainable management of Antarctic resources demands new policies to prevent environmental degradation from unmonitored prospecting. Additionally, as these assets belong to the common heritage of humanity, the international community must clarify ethical concerns surrounding ownership and benefit-sharing to ensure equitable access and environmental protection [77].

## 9. Future Perspectives: The Omics Revolution in a Warming Cryosphere

For decades, the Antarctic continent was viewed as a biologically isolated system. However, the rapidly warming cryosphere has shattered this paradigm. As ice sheets retreat and permafrost thaws, previously sequestered organic carbon is released, triggering distinct shifts in microbial community structures. Recent research in 2025 warns that these shifts are not just taxonomic but functional: hydrocarbon contamination, even after 40 years, can severely inhibit trace gas oxidation (e.g., atmospheric H_2_ scavenging), a critical ecosystem service that stabilizes the polar climate [78].

### 9.1. Bioremediation in a Warming Antarctica

Seasonal thawing and permafrost degradation increase the mobilization of hydrocarbons and other legacy contaminants, enhancing their bioavailability and facilitating their transfer to highly vulnerable coastal and marine systems [20]. This process expands the spatial extent of environmental impacts and alters soil redox conditions and hydrological dynamics, thereby constraining the effectiveness of natural attenuation processes and any remediation intervention [79].

An emerging risk is the release of methane (CH_4_) from soils, sediments, and subglacial environments that harbor latent methanogenic communities. Although Antarctica has historically been a minor source of CH_4_ compared to the Arctic, warming and increased availability of organic carbon may activate these processes, generating positive climate feedbacks [80]. Managing this risk requires considering methane as an integral component of impact assessments, alongside traditional chemical contaminants [81]. Methanotrophs play a critical role as biological sinks for methane [82]. Psychrotolerant and psychrophilic communities, through the activity of methane monooxygenase, can oxidize CH_4_ prior to its release to the atmosphere, functioning as natural biogeochemical filters in soils and sediments [83]. Moderate warming may even enhance their activity, provided that adequate oxygenation and moisture conditions are maintained, thereby opening opportunities for biostimulation strategies aimed at strengthening native microbial functions without introducing exogenous organisms [84].

Methanotroph-based biofilters represent a promising tool for the targeted mitigation of point-source CH_4_ emissions associated with human infrastructure or zones of active thaw [85]. Designed under the principle of minimal environmental intervention, these systems can efficiently intercept and oxidize low-concentration methane [86]. However, their application in Antarctica must be approached with extreme caution, prioritizing the use of autochthonous consortia, assessing potential ecological risks, and strictly adhering to the Antarctic Treaty System. Overall, integrating contaminant bioremediation with microbial methane mitigation is essential to address the challenges posed by a warming Antarctica in a scientifically robust and environmentally responsible manner [87].

### 9.2. Expanding the Genetic Toolkit: Beyond alkB

Traditional studies have focused heavily on the *alkB* gene for alkane degradation. However, metagenomic surveys published in late 2024 and 2025 have revealed a broader arsenal of catabolic potential. The identification of *almA* and *ladA* genes in genera like *Marinomonas* and *Alteromonas* explains how Antarctic consortia degrade long-chain alkanes (C_20_) that were previously thought to be recalcitrant [88]. Integrating these novel markers into monitoring programs will allow for a more accurate assessment of “natural attenuation” potential in aged spills.

### 9.3. Unlocking the “Microbial Dark Matter”

The future of bioremediation lies in multi-omics technologies. Metagenomics and metatranscriptomics allow us to access the functional potential of the “unculturable majority”. As detailed in Table 5, recent studies have successfully mined Antarctic metagenomes to identify genes expressed only under specific stress conditions, such as high salinity or oxidative stress [89]. These data suggest that successful bioaugmentation requires not just hydrocarbon-degrading capability but a suite of “stress-response” genes (*cpnA*, czcA) to ensure survival in the hostile Antarctic soil.

### 9.4. Climate-Smart Bioremediation

**Figure 4:**
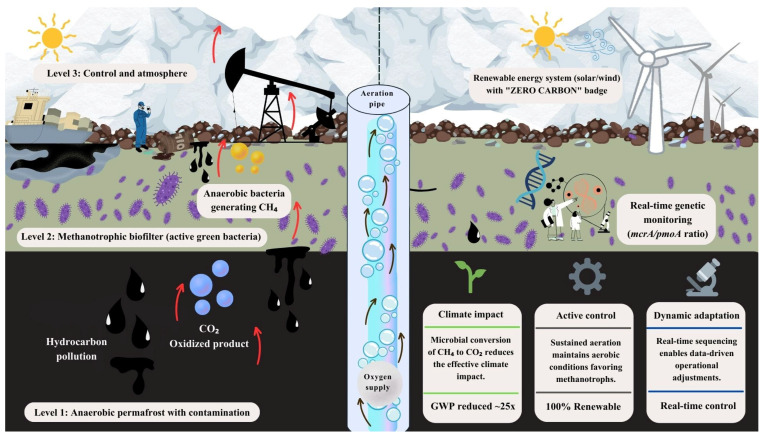
Climate-smart bioremediation framework in contaminated Antarctic soils.

Finally, the intersection of bioremediation and climate change cannot be ignored. Thawing permafrost creates anaerobic microenvironments in which hydrocarbon degradation may stimulate methanogenic activity, potentially increasing methane emissions. Consequently, future remediation strategies should aim to balance contaminant removal efficiency with greenhouse gas mitigation. One promising approach involves the incorporation of aerobic methanotrophs as a biological “biofilter” to oxidize methane before its release into the atmosphere, thereby preventing remediated sites from becoming net carbon sources [78] (Figure 4).

To support this strategy, real-time monitoring of microbial functional dynamics becomes essential. In this context, the ratio of *mcrA* to *pmoA* genes can serve as a molecular indicator of the balance between methane production and oxidation processes. The *mcrA* gene (methyl coenzyme M reductase subunit A) is widely recognized as a biomarker for methanogenic archaea, whereas the *pmoA* gene (particulate methane monooxygenase subunit A) is commonly used to detect methanotrophic bacteria involved in methane oxidation. Therefore, the *mcrA*/*pmoA* ratio provides insight into the relative abundance and activity potential of methane-producing versus methane-oxidizing microorganisms, enabling performance evaluation and facilitating data-driven adjustments to maintain environmental conditions that favor methane mitigation.

## 10. Conclusions

The remediation of hydrocarbon-contaminated soils in Antarctica has transitioned from an empirical practice based on trial-and-error biostimulation to a precision science guided by molecular ecology. This review highlights that while temperature remains a governing thermodynamic constraint, the primary bottleneck for biodegradation is the bioavailability of aged, hydrophobic contaminants. The shift toward microbial consortia over single-strain applications addresses the frequent failure of isolates to compete with indigenous communities. Indeed, synergistic partnerships between fungi and bacteria yield superior results, as fungal laccases break complex aromatics into intermediates that bacteria can then fully mineralize. Furthermore, recent metagenomic studies (2024–2025) have begun to unlock the potential of microbial ‘dark matter,’ identifying critical genes such as *almA* and *ladA*, which confirm the existence of metabolic machinery for degrading heavy alkanes under extreme cold. Finally, future bioremediation strategies must be integrated with climate change considerations; in thawing permafrost, hydrocarbon degradation can trigger methanogenesis; thus, methanotrophic ‘biofilters’ are needed to avoid significant sources of greenhouse gas emissions. Future research must pivot towards “system bioremediation,” integrating metatranscriptomics with field engineering to optimize nutrient stoichiometry and mass transfer. Ultimately, preserving the Antarctic legacy requires developing “climate-resilient” biotechnologies capable of functioning in a rapidly changing cryosphere.

## Figures and Tables

**Table 1:** Key hydrocarbon-degrading bacteria isolated from Antarctica (2018–2024) and their adaptation mechanisms.

Organism	Isolation Site	Targeted Substrate	Adaptation Mechanism/Key Enzymes	Reference
*Pseudomonas fildesensis* *Janthinobacterium lividum*	Fildes Peninsula	Diesel (C12–C26)	Production of glycolipid biosurfactants; high emulsification index (E24 > 60%) at low temperatures. Cold-active lipase production and significant reduction in surface tension	[14]
*Rhodococcus*	Ross Sea Region	Phenol & Aromatics	Expression of catechol 1,2-dioxygenase and catechol 2,3-dioxygenase active at 4 °C.	[30]
*Arthrobacter* sp.	King George Island	Crude Oil	Membrane lipid modification (increased unsaturated fatty acids) to maintain fluidity	[31]
*Sphingobium* sp.	Antarctic Soil	Carbazole (N-heterocyclic)	Identification of car genes encoding terminal oxygenases specific for recalcitrant rings	[32]

**Table 2:** Enzymatic profile and degradation potential of Antarctic fungi (2019–2023).

Organism Type	Species	Targeted Substrate	Key Enzymatic/Metabolic Mechanism	Reference
**Filamentous Fungus**	*Aspergillus glaucus*	Naphthalene, Anthracene	Production of phenol monooxygenase and catechol 1,2-dioxygenase.	[47]
**Filamentous Fungus**	*Penicillium* CHY-2	Diesel, Aliphatic (C_10_–C_28_)	High laccase activity and extracellular oxidative stress response.	[45]
**Yeast**	*Pichia caribbica*	Diesel, n-alkanes	High production of esterases and lipases; emulsification of hydrophobic phases.	[48]
**Filamentous Fungus**	*Pseudogymnoascus pannorum*	Aged Diesel	Psychrophilic growth; dominates in hydrocarbon-impacted permafrost.	[49]

**Table 3:** Comparison of in situ and ex situ bioremediation strategies in Antarctica (2003–2017).

Strategy	Advantages	Disadvantages	Case Study Results	Reference
**In situ** Microcosms and biostimulation in Antarctic soils	- Maintains the soil in its original environment. - Uses indigenous microorganisms adapted to cold conditions. - Minimal physical disturbance to the ecosystem.	- Extreme environmental conditions hinder biodegradation. - Limited control of variables (temperature, moisture, nutrients). - Slower or more variable results among sites	- Experiments using contaminated soil microcosms showed that the indigenous microbial flora degraded a significant fraction of hydrocarbons (~35% higher than the control), and bioaugmentation with a psychrotolerant strain increased hydrocarbon removal up to ~75%.	[59]
**Ex situ** On-site biopiles with biostimulation (geomembranes)	- Allows control of nutrients (N and P) and physical conditions to enhance degradation. - Higher hydrocarbon removal efficiency in a shorter time. - Experimental design enables improved monitoring and management	- Requires soil excavation and biopile construction, increasing logistical demands. - Greater physical intervention in the environment. - Higher logistical and installation costs.	- A field study at Carlini Station showed that, with optimized nitrogen and phosphorus biostimulation, biopiles achieved ~75.79% hydrocarbon removal in 50 days, compared to ~49.54% in unstimulated control biopiles	[60]
**Large-scale ex situ** Biopiles with soil management and reuse	- Enables rehabilitation of large soil volumes with significant contaminant removal. - Remediated soil can be reused for infrastructure development	- Long-term process (years). - Logistical complexity in remote areas and under extreme climatic conditions.	- In the first large-scale biopile project in Antarctica, active remediation reduced contaminant levels by a factor of four over five years, with 370 t of remediated soil reused for infrastructure purposes	[61]

**Table 4:** Efficiency of in situ and ex situ field trials in Antarctica (2000–2021).

Location/Station	Treatment Strategy	Nutrient Source/Amendment	Target Contaminant	Removal Efficiency	Reference
Casey station (Australia)	Ex situ Biopile	Ammonium chloride + Superphosphate (Optimized C:N:P)	SAB diesel	75% (51 days)	[61]
Carlini station (Argentina)	Ex situ Biopile	Commercial fertilizer (N:P:K) + Geomembrane		75% (40 days)	[60]
		Osmocote (Slow-release N-P)		76% (45 days)	[60]
Signy Island (UK)	Bioaugmentation	Indigenous *Pseudomonas* sp. *consortium*	Lubricating oil	81–86% (45 days)	[64]
Marambio base (Argentina)	In situ Biostimulation	Urea + Phosphate	Jet Fuel (JP-1)	50% (50 days)	[65]
McMurdo station (USA)	Bioventing	Air injection + Warm air	TPH	Variable (Limited by temp)	[11]

**Table 5:** Key functional genes and metabolic pathways identified in Antarctic soils via metagenomics and genomic screening (2020–2025).

Year	Target Gene	Enzyme/Protein	Function in Remediation	Associated Genera	Reference
2025	*almA*	Flavin-binding monooxygenase	Degradation of recalcitrant long-chain alkanes C20 in aged spills.	*Marinomonas*, *Alteromonas*	[90]
2025	*hhyL*	High-affinity hydrogenase	Oxidation of atmospheric H2 (trace gas scavenging); inhibited by oil spills.	*Actinobacteria*	[78]
2024	*nahAa*	Naphthalene 1,2-dioxygenase	Initial oxidation of PAHs under specific salinity stress conditions.	*Pseudarthrobacter*, *Pseudomonas*	[91]
2023	*carAa*	Carbazole 1,9a-dioxygenase	Cleavage of N-heterocyclic aromatic rings (carbazole) in surface soils.	*Sphingobium*	[32]
2022	*alkB*	Alkane-1-monooxygenase	Correlation between gene copy number and seasonal water table fluctuations.	*Rhodococcus*, *Pseudomonas*	[26]
2021	*nidA*	Pyrene dioxygenase (alpha sub)	Degradation of high-molecular-weight PAHs (4 rings) in rhizosphere soils.	*Mycobacterium*	[92]
2020	*czcA*	Co-Zn-Cd efflux protein	Heavy metal resistance in co-contaminated diesel soils.	*Ralstonia*, *Burkholderia*	[28]

## Data Availability

No new data were created or analyzed in this study. Data sharing is not applicable to this article.

## Abbreviations

AlkB	Alkane monooxygenase involved in the degradation of alkanes
*almA*/*ladA*	Specific genes for the degradation of long-chain alkanes
BTEX	Benzene, toluene, ethylbenzene, and xylene (monocyclic aromatic compounds)
C:N:P	Carbon–nitrogen–phosphorus ratio (nutrient balance)
CABS	Cold-active biosurfactants
CAZymes	Cold-active enzymes
E24	Emulsification index at 24 h
LiP	Lignin peroxidases
MnP	Manganese peroxidases
*nahAc*/*ndoB*	Genes encoding Antarctic variants of RHDs
PAHs	Polycyclic aromatic hydrocarbons
RHDs	Ring-hydroxylating dioxygenases
SAB	Special Antarctic blend (diesel fuel)
TCA	Tricarboxylic acid cycle (Krebs cycle)
TPHs	Total petroleum hydrocarbons
